# Autofluorescence Detection Method for Dental Plaque Bacteria Detection and Classification: Example of *Porphyromonas gingivalis*, *Aggregatibacter actinomycetemcomitans*, and *Streptococcus mutans*

**DOI:** 10.3390/dj9070074

**Published:** 2021-06-22

**Authors:** Yung-Jhe Yan, Bo-Wen Wang, Chih-Man Yang, Ching-Yi Wu, Mang Ou-Yang

**Affiliations:** 1 Institute of Electrical and Control Engineering, National Yang Ming Chiao Tung University, 1001 University Road, Hsinchu City 30010, Taiwan; jerryyan.eed02g@nctu.edu.tw; 2 Department of Electrical and Computer Engineering, National Yang Ming Chiao Tung University, 1001 University Road, Hsinchu City 30010, Taiwan; muziktsubasa@gmail.com; 3 Department of Laboratory Medicine, National Taiwan University Hospital Hsin-Chu Branch, No. 25 Ln. 442 Jingguo Road, Hsinchu City 30010, Taiwan; yang-mann@yahoo.com.tw; 4 Institute of Molecular Medicine and Biochemical Engineering, National Yang Ming Chiao Tung University, 1001 University Road, Hsinchu City 30010, Taiwan; 5 Institute of Oral Biology, National Yang Ming Chiao Tung University, 155 Linong Street, Taipei City 11221, Taiwan; cywu3@ym.edu.tw

**Keywords:** plaque detection, dental plaque, autofluorescence, *Porphyromonas gingivalis*, *Aggregatibacter actinomycetemcomitans*, *Streptococcus mutans*

## Abstract

The use of fluorescence spectroscopy for plaque detection is a fast and effective way to monitor oral health. At present, there is no uniform specification for the design of the excitation light source of related products for generating fluorescence. To carry out experiments on dental plaque, the fluorescence spectra of three different bacterial species (*Porphyromonas gingivalis*, *Aggregatibacter actinomycetemcomitans*, and *Streptococcus mutans*) were measured by hyperspectral imaging microscopy (HIM). Three critical issues were found in the experiments. One issue was the unwanted spectrum generated from a mercury line source; two four-order low-pass filters were evaluated for eliminating the unwanted spectrum and meet the experimental requirements. The second issue was the red fluorescence generated from the microscope slide made of borosilicate glass; this could affect the observation of the red fluorescence from the bacteria; quartz microscope slides were found to reduce the fluorescence intensity by about 2 dB compared with the borosilicate slide. The third issue of photobleaching in the fluorescence of the *Porphyromonas gingivalis* was studied. This study proposes a method of classifying three bacteria based on the spectral intensity ratios (510/635 and 500/635 nm) under the 405 nm excitation light was proposed in this study. The sensitivity and specificity of the classification were approximately 99% and 99%, respectively.

## 1. Introduction

Dental plaque is composed of tiny food particles, mass bacteria, and waste products of bacteria on the tooth surface or other surfaces in the oral cavity. The build-up of dental plaque often causes gingivitis, periodontitis, and caries. According to a Health Promotion Administration survey in 2012, the prevalence of caries in Taiwan exceeded 88%, and the proportion of periodontitis and gingivitis, which are associated with periodontal disease, reached 99% [1]. If gingivitis, periodontitis, and caries diseases are not properly treated at an early stage, they cause tooth loss and accompany an increasing failure rate for dental implants. Due to inflammation of the mucous membrane and bone loss accompanying periodontal disease, implanting a denture is like building a house on a hollow foundation, and it will finally fall apart. Dental plaque build-up is normally caused by poor oral hygiene. As the plaque biofilm matures, the secreted acidic substances erode the teeth, decalcify the tooth surface, and form caries. In addition, lipopolysaccharide, found in the outer membrane of Gram-negative bacteria, might induce the loss of periodontal ligament and alveolar bone, causing teeth to shake and fall out. Moreover, some bacteria produce proteases to degrade host tissues. The two pathogenic factors mentioned above will continue to pose harm even if the bacteria are killed.

Bacteria can be divided into two broad categories: Gram-positive bacteria and Gram-negative bacteria. The Gram staining classification method makes the Gram-positive bacteria remain purple, while Gram-negative bacteria are stained pink or red in counterstain [2,3]. Considerable research over several decades has led to the identification of *Porphyromonas gingivalis* (*PG*), a Gram-negative anaerobic bacterium, as the major pathogenic bacterium that contributes to the development of periodontal disease [4]. The *PG* was found in 87.75% of subgingival plaque samples from chronic periodontitis patients. *Aggregatibacter actinomycetemcomitans* (*AA*), a Gram-negative facultative anaerobic bacterium, is highly associated with localized aggressive periodontitis. It has been further implicated in various systemic diseases, including infectious endocarditis, brain abscesses, and chest wall abscesses [5]. *Streptococcus mutans* (*SM*), a Gram-positive facultative anaerobic bacterium, plays a major role in tooth decay. On the basis of the above reasons, the *AA*, *PG*, and *SM* were the target bacteria species in the current study.

Although oral cleaning procedures can easily remove dental plaque, oral hygiene is often neglected. Various dental plaque disclosing methods have been developed to assess dental plaque levels. The traditional method is to rinse the mouth with a disclosing solution containing red pigment and erythrosine. Absorption plays an important part during this process because erythrosine preferentially binds to plaque rather than teeth. However, the result of this method is affected by the staining time and oral muscle movements. It does not have intuitive or quantitative characteristics; moreover, residual pigments can cause esthetic problems. In recent years, fluorescent imaging technology has been widely used in biomedical detection. The endogenous fluorophores were irradiated by ultraviolet light and emit visible light. The energy difference between the absorption wavelength and fluorescence wavelength is known as the Stokes shift [6]. This fluorescence characteristic can be used to detect dental plaque. The traditional method and fluorescent imaging technology are compared in Table 1.

The red fluorescence has been found in a biofilm that might be originated from porphyrins produced during bacterial metabolites [7]. The red fluorescence has been used to detect dental caries, oral malodor, and dental plaque [7,8,9,10,11,12,13,14,15,16]. Coulthwaite et al. observed the fluorescence of plaque on dentures using 366 nm excitation light sources [7]. Rechmann et al. discriminated plaque and gingival inflammation based on a SOPROCARE camera system and a 450 nm excitation light source [8]. Joseph et al. detected dental plaque in situ based on laser-induced autofluorescence spectroscopy equipped with a 404 nm excitation light source [9]. Liu et al. detected in-vitro dental plaque based on red fluorescence with a 405 nm excitation light source [10]. The wavelength of the excitation light sources in these studies seems inconsistent. Furthermore, bacteria species might produce different fluorophores that have individual fluorescent spectra induced by the specific wavelengths of excitation light sources. Thus, this study aims to find the optimal fluorescence stimulation band and find the significant spectral band in three kinds of common bacteria, including *PG*, *AA*, and *SM*. Caries could be induced by *SM*, and periodontal disease could be induced by *PG* and *AA*; the identification of bacteria species in dental plaque might help to prevent people from developing caries, periodontal disease, and subsequent derivation of systematic disease. Thus, the goal of this study is to identify three bacteria species based on light-induced autofluorescence spectroscopy.

## 2. Materials and Methods

### 2.1. Hyperspectral Microscopy System

**Figure 1:**
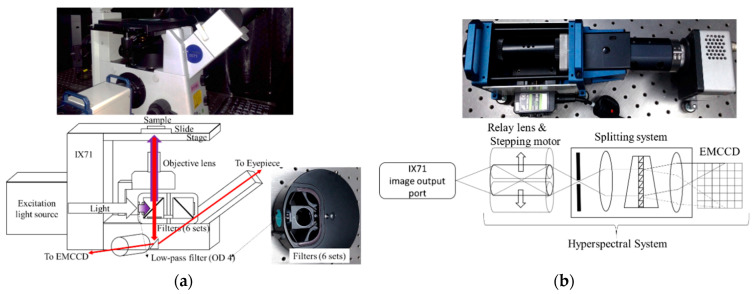
(**a**) Inverted microscope structure in the hyperspectral microscopy system. (**b**) Hyperspectral system equipped on the hyperspectral microscopy system.

The hyperspectral microscopy system (HMS) was developed in a previous study to collect the fluorescence spectra of bacteria [17]. The HMS is mainly composed of a microscopy system and a hyperspectral imaging system (HIS). The microscopy system consists of an Olympus IX-71 inverted microscope, a CCD camera, and two emission light sources, including a halogen source and a mercury source (Figure 1a). The microscope provides an eyepiece, with additional image output ports on the left and right sides of the observation window. The left-side output port is connected to the HIS, and the right-side output port is connected to the CCD. The halogen source was used in the experiment to supply excitation lights in different bands. The excitation band was selected according to the specified excitation filter. The excitation light is reflected by the dichromatic mirror and passes through the objective lens (OBL) to irradiate a sample. After irradiation, the sample is excited and emits fluorescence. The fluorescence passes through the OBL and the emission filter. The beams splitter 1 splits the fluorescence into two parts. One part is passed to the CCD or the eyepiece and the other to the HIS. The HIS is composed of a relay lens, a stepping motor, a spectrometer (Specim V10E), and an electron-multiplying charge-coupled device (EMCCD) camera (Anode Luca R604). The relay lens is composed of multiple symmetrical lenses to transfer images from one side to the other. The image is laterally and vertically inverted. The stepping motor controls the coil current and magnetizes the opposite rotor at a certain angle to control the position of the relay lens. It can achieve the advantage of precise rotation. The system scans the target by moving the relay lens, allowing the target to be measured without moving the target or the system. The splitting system decomposes the input light into spectra from 400 to 1000 nm. It includes all visible light and part of the UV and near-infrared light. The EMCCD has an electronic gain function that allows the charge to multiply through the gain register and amplify the weak signal to facilitate the collection of the weak fluorescent signals. The spectral resolution of the HIS is about 2.8 nm, and the spatial resolution is about 30 μm × 10 μm. Accordingly, the HIS can collect the bacteria-scale fluorescent image over hundreds of bands.

### 2.2. Specific Consideration for Eliminating the Excitation Light

A few narrowband wavelengths in the mercury line were used as the excitation light in the experiments. The 365 nm filter was installed in front of the mercury light source, and five narrow band-pass filters with center wavelengths of 375, 394, 405, 420, and 430 nm were equipped. Each filter had a photon attenuation (absorption and scattering) or optical density (OD) that resulted in an exponential decay of intensity of any given optical field, as it penetrated deep into tissue and limited the penetration depth achievable for deep tissue imaging.

**Figure 2:**
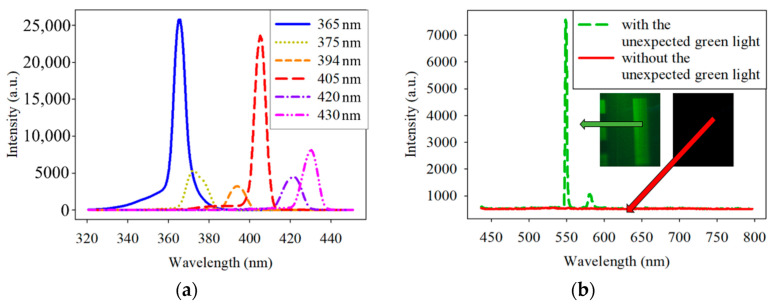
(**a**) Spectrum of the excitation light source filtered by 365-, 375-, 394-, 405-, 420-, and 430 nm band-pass filters. (**b**) Spectrum of the excitation light source with and without two 450 nm (OD = 4) low-pass filter. OD is the order of the penetration rate.

Unexpectedly, the EMCCD-acquired image contained green light. Therefore, all the spectra of the excitation lights filtered by the six band-pass filters were checked. The results in Figure 2a show that the filters were correctly designed because each filter has only one narrow peak at the specified band. The penetration rate α is the emitted light divided by the incident light, and it satisfies Equation (1). To counteract the intensity of the undesired green light, two 450 nm low-pass filters with the order (OD) of 4, with the same effect of one 450 nm low-pass filters with the order (OD) of 8, were positioned in front of all the narrowband filters. The spectra of the excitation light source coupled with and without the 450 nm low-pass filter are shown in Figure 2b. These low-pass filters dramatically reduced the intensity of the unexpected green light.

(1) $$\alpha =10^{-\mathrm{OD}}$$

### 2.3. Object Slide of Microscope Necessary for Autofluorescence Measurement

**Figure 3:**
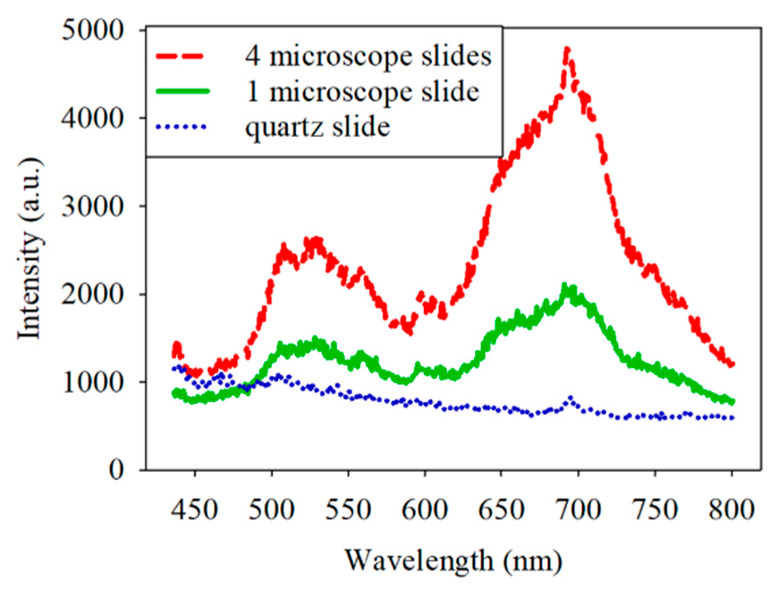
Spectrum obtained with the four borosilicate slides, one borosilicate slide, and one quartz slide.

At the beginning of the experiment, the spectrum of each sample was characterized by a weak red fluorescence with a peak wavelength of about 650−700 nm. To determine the source of the red fluorescence, the sample and filter sets were removed first, and only one or four microscope slides were equipped on the microscope. The corresponding spectra are shown in Figure 3. The red intensity of the spectrum was higher with the four microscope slides than with the one microscope slide. Notably, the general microscope slide is made of borosilicate glass, which was found to generate red fluorescence when excited at 445 nm [18]. Therefore, it is reasonable to infer that the microscope slide generated the red fluorescence. It was essential to eliminate the red fluorescence because its intensity is close to that of the bacteria and could affect the analysis. Quartz slides presented an alternative to borosilicate glass. Figure 3 displays the spectrum obtained with the quartz slide. It shows a much lower intensity of red fluorescence compared to borosilicate glass. The intensity at a 700 nm wavelength for quartz is reduced by 2 dB compared to borosilicate glass. For accurate measurements, the subsequent experiments used quartz slides instead of borosilicate glass.

### 2.4. Culture the Bacteria

Three species of bacteria (*PG*, *AA* and *SM*) were obtained from Bioresource Collection and Research Center, Hsinchu City, Taiwan. The bacteria were stored in a stock with TYHK medium composed of trypticase soy broth (30 g/L), yeast extract (5 g/L), hemin (1 μg/mL), vitamin K3 (1 μg/mL), and brain heart infusion with 10% glycerol. The bacteria were cultured and stocked in the biosafety level two (BSL-2) laboratory at National Taiwan University Hospital Hsin-Chu Branch, Hsinchu City, Taiwan. The bacteria were cultured in a suitable environment, Petri dish, and medium. Specifically, CDC anaerobic blood agar was used to facilitate the cultivation of the *PG* and the *AA* at 37 °C in an anaerobic tank, and tryptic soy agar with 5% sheep blood agar was used to facilitate the cultivation of the *SM* at 37 °C in an aerobic tank. The two agars were manufactured by Dr. Plate Co. The bacteria were first stocked in Bacterial Freezing medium tubes. After removing some of each bacterial species from the Bacterial Freezing medium tubes using sterile loops, it was smeared on the four regions of the agar using the streaking method. *PG* and *AA* were cultured for 2–3 weeks and *SM* was cultured for 3–4 days before the exiting and scanning processes using the HMS. In the experiment, six plates of *PG*, six plates of *AA*, and 15 plates of *SM* were cultured.

## 3. Results and Analysis

### 3.1. Photobleaching of PG

**Figure 4:**
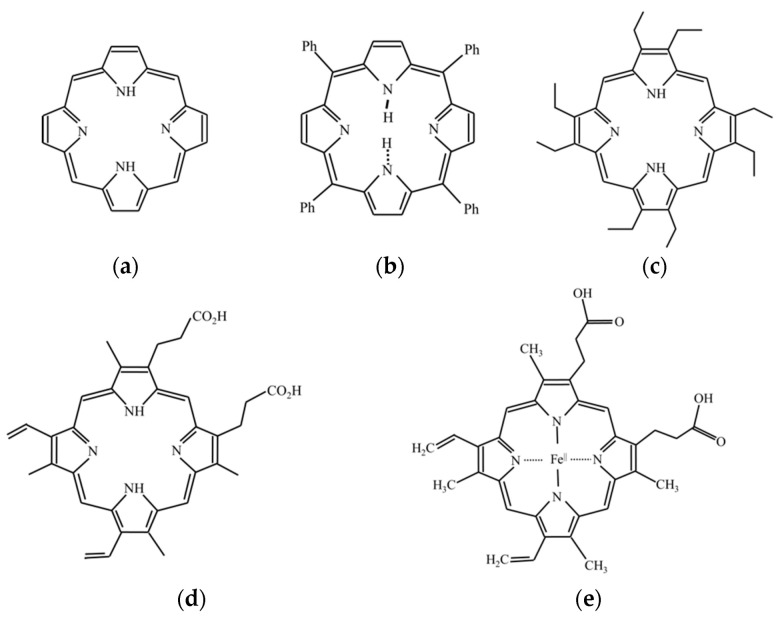
(**a**) Chemical structure of porphyrin, (**b**) tetraphenylporphyrin, (**c**) octaethylporphyrin, (**d**) protoporphyrin IX, and (**e**) heme.

Photobleaching describes the photochemical alteration of a fluorophore in such a way that it permanently loses its ability to fluorescence [19,20]. The phenomenon, though not yet fully understood, is irreversible because a covalent bond in a fluorophore molecule that transits from the singlet state to the triplet state. Some fluorescent materials undergo bleaching as soon as they are excited, while some may experience thousands or millions of fluorescent cycles before bleaching [19,20]. The loss of fluorescence in photobleaching follows an exponential decay. The protoporphyrin IX was detected in *PG* [21], and the fluorescent spectrum of the porphyrins was consistent with the fluorescent spectrum of the PG in this study [22,23]. Thus, the protoporphyrin IX might be the main contribution of the fluorescent spectrum in *PG*. Figure 4 shows the porphyrin structure and the structures of parent porphyrin [23,24,25,26,27].

One feature of exponential decay is the half-life (*t*_1/2_), which refers to the time it takes for a certain concentration of a substance to be reduced to half of its initial concentration after a certain reaction. The *t*_1/2_ is defined as follows:

(2) $$N\left(t\right)=N_{0}\;e^{-\lambda t},$$

(3) $$t_{1/2}=\frac{ln2}{\lambda },$$

where *N*_0_ is the initial intensity, *N*(*t*) is the intensity at time *t*, and *λ* is the reaction rate constant. As the fluorescence is generated not only from porphyrin but also from bacteria and other substances, the offset of parameter *M* should be added as compensation in Equation (4).

(4) $$N\left(t\right)=M+N_{0}\;e^{-\lambda t}.$$

The fluorescence decay complicates the quantitative analysis of the hyperspectral measuring data. The different positions of the sample must be measured at different times because the hyperspectral system measures a narrow slot area of a sample at one time, referred to as the line scan, and the sample area is normally larger than the slot of the HMS. Furthermore, because the whole sample was excited at the same time in the experiments, fluorescence decay may also start at the same time. These reasons caused the variance of the spectrum measurement position with increased durations of bleaching.

**Figure 5:**
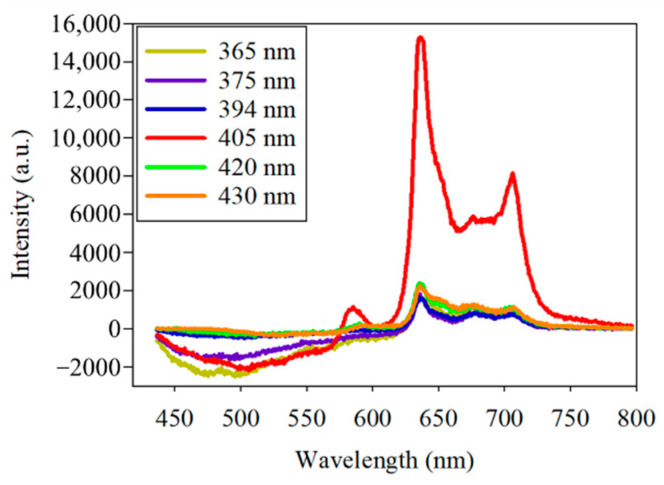
Spectra of the fluorescence of *Porphyromonas gingivalis* by six excitation lights, including the center wavelengths of 365, 375, 394, 405, 420, and 430 nm.

Photobleaching occurred in the initial fluorescence experiment of *PG*. To further observe the fluorescence decay exhibited by *PG* at different excitation wavelengths, two plates of *PG* and six excitation wavelengths, including 365, 375, 394, 405, 420, and 430 nm, were used. At first, the bacteria-free quartz slide was scanned to measure the background signal. Some *PG* was removed from the Petri dish using a sterile loop and was smeared onto the quartz slide for excitation and scanning. To observe the fluorescence decay under the same intensity of the excitation wavelengths for the two *PG* samples, the same position of the *PG* samples in a slot range was scanned several times for a fixed scanning duration and exposure time. The fluorescence emission spectra of *PG* showed a peak wavelength at about 635 nm at the different excitation wavelengths (Figure 5). The highest fluorescence intensity was observed at the 405 nm excitation wavelength. The photobleaching of *PG* at the 405 nm excitation wavelength, as seen in the experimental results, was further analyzed.

**Figure 6:**
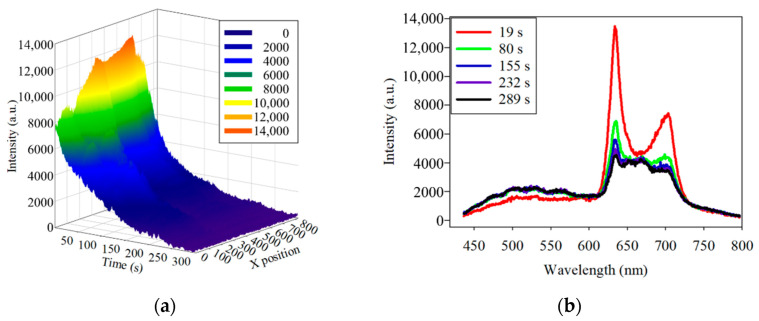
(**a**) three-dimensional plot of the fluorescence decay of *Porphyromonas gingivalis* at 635 nm under 405 nm excitation light. (**b**) Spectra of the fluorescence decay at the same position at different times. To determine the consistency of the photobleaching, the fluorescence-decay curves of the *PG* samples were fitted to an exponential curve. Half of the difference between the maximum and minimum values of the decayed fluorescence intensities was considered as *t*_1/2_. Based on Equation (3), the λ can be derived by the known *t*_1/2_. The simulated curves were calculated and plotted according to Equation (4) by MATLAB™. The fluorescence decay of two *PG* samples at 635 nm at two line positions fitted to the exponential curve is shown in Figure 7.

The photobleaching of the *PG* samples at 405 nm excitation light is shown in Figure 6a. The fluorescence intensity at about 635 nm wavelength was decaying when the HMS was continuously scanning. The fluorescence spectra at the line position of 552 and at different times, including 19, 80, 155, 232, and 289 s, are further shown in Figure 6b. The spectra illustrate that the fluorescence of *PG* decayed at the wavelengths of 635 and 702 nm.

**Figure 7:**
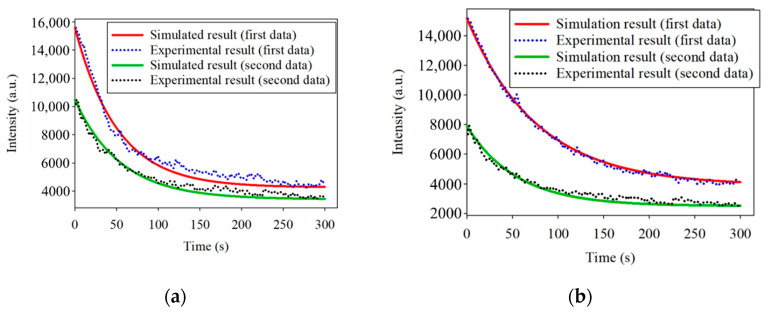
(**a**) Simulated and experimental results of the photobleaching at 635 nm excited by 405 nm at the spatial position of 552; (**b**) Simulated and experimental results of the photobleaching at 635 nm excited by 405 nm at the spatial position of 488.

The simulated curves in Figure 7a,b are similar to the experimental curves. However, *t*_1/2_ of one sample at the different spatial positions was not identical. This phenomenon may be caused by variations in the thickness of the bacteria at different spatial positions. The thickness variation may lead to a variation in the fluorescence processing time. Considering that the bacteria in the samples were excited from the bottom to the top, the fluorescence reaction also started from the bottom to the top. Although the whole sample was uniformly excited, the different thicknesses of the bacteria led to differences in the fluorescence *t*_1/2_ values. Furthermore, the reason for the thickness variation is the limited control over the number and uniform thickness of the bacteria in the different positions during the experiments. If there were a way to stabilize the number of bacteria, the experiments could be further carried out to obtain various light intensities corresponding to various quantities.

**Figure 8:**
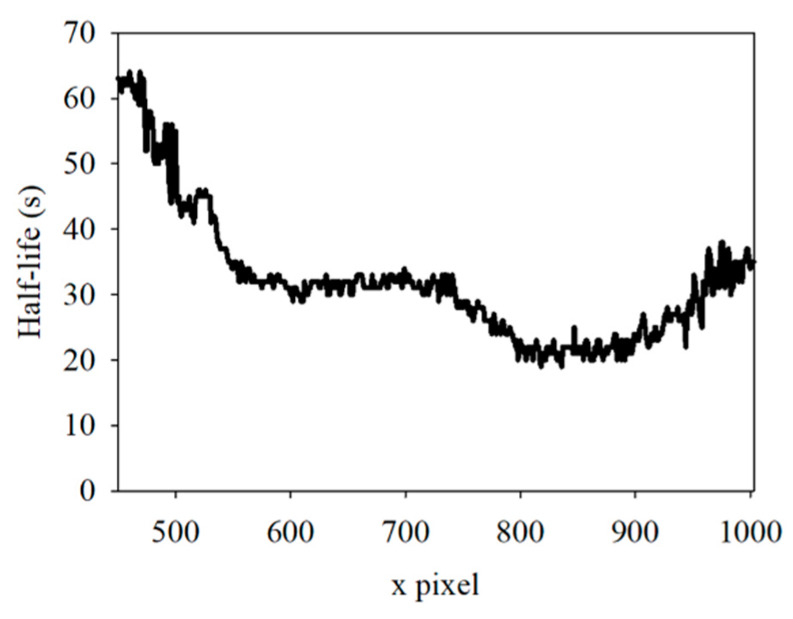
Half-life in spatial position from 450 to 1003.

Maturely, the sample has continuous thickness variation across the positions, and the excitation light intensity is continuous in a nearby location. Therefore, the *t*_1/2_ of the fluorescence must continuously vary with the positions. To prove this phenomenon, the *t*_1/2_ of the first sample in the region from 450 to 1003 was calculated and plotted in Figure 8. The results showed that the *t*_1/2_ was a continuous curve, making the inference more convincing.

### 3.2. Classification of PG/AA/SM

**Figure 9:**
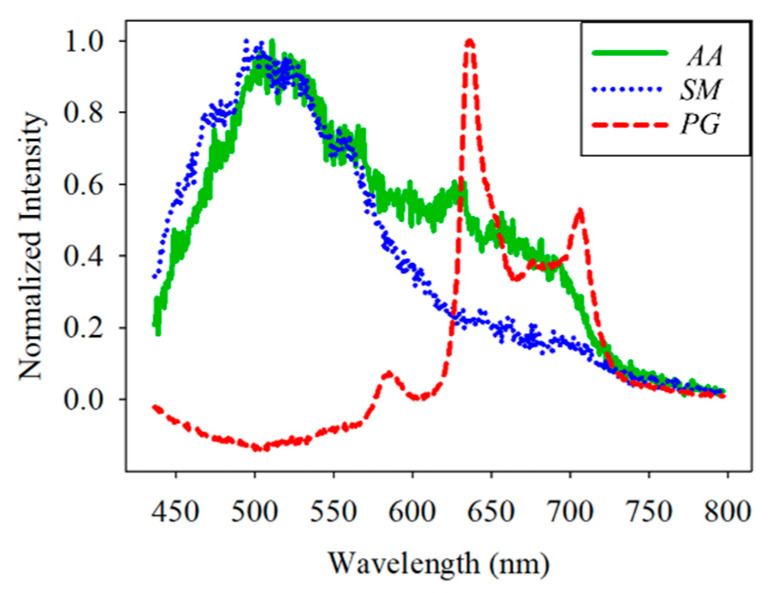
Normalized spectral curve of *Porphyromonas gingivalis* (*PG*), *Aggregatibacter actinomycetemcomitans* (*AA*), and *Streptococcus mutans* (*SM*).

**Figure 10:**
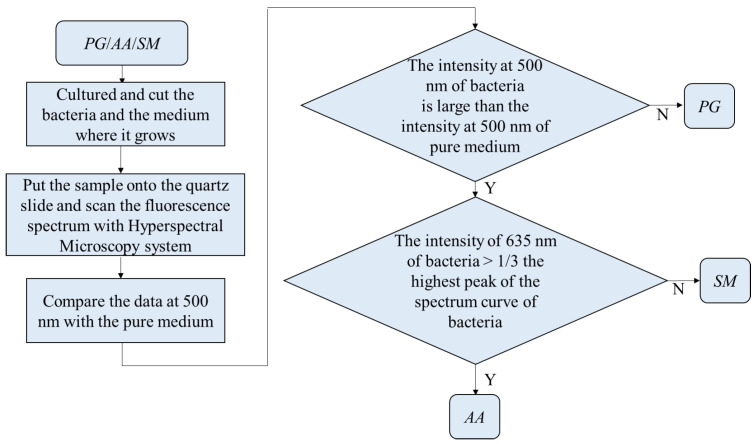
Flow chart of the classification in *Porphyromonas gingivalis* (*PG*), *Aggregatibacter actinomycetemcomitans* (*AA*), and *Streptococcus mutans* (*SM*).

**Figure 11:**
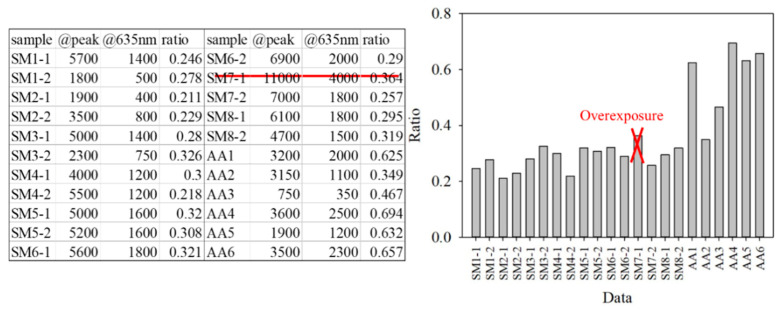
Peak intensity, the intensity at 635 nm, and the ratio of the intensity at 635 nm to the peak intensity of every group.

For the classification, the bacteria and part of the agar containing the bacteria were placed on the quartz slide and scanned. Part of the bacteria-free agar was measured as the background. The spectrum data were normalized against the maximum intensity of each spectrum. The normalized fluorescence spectra of *PG*, *AA* and *SM* at 405 nm excitation light are shown in Figure 9. After subtracting the background, the spectrum of *PG* had a valley with a negative value at about 500 nm. This is due to the composition variation in the agar during culturing. *AA* and *SM* had a positive value; this was significantly different from *PG*. Thus, the negative peak at 500 nm was used to differentiate *PG* from the others. The fluorescent spectrum of *AA* and *SM* had the same peak at 500 nm but differed at 635 nm. To classify *AA* and *SM*, the ratio of intensity at 635 ± 1 nm to the highest peak intensity at around 510 ± 1 nm of *AA* and 500 ± 1 nm of *SM*, respectively, was considered to identify the corresponding bacteria. Figure 10 shows the flow chart of the classification criteria. For each bacterial species, the ratio of one point was calculated as a simple reference. The results are shown in Figure 11. It is worthy of mentioning that the peak intensity of the experimental raw data was larger than 10,000; overexposure occurred, and the data were excluded from the analysis. In Figure 11, the SM7-1 had a peak intensity larger than 10,000. The SM7-1 data were excluded because the EMCCD was overexposed to measure this data. A total of 6 and 15 groups of *AA* and *SM* were recruited, respectively. The ratio at approximately 0.33 was the standard to distinguish between *AA* and *SM*. If the ratio was higher than 0.33, the sample was identified as *AA*. If the ratio of the sample was lower than 0.33, the sample was identified as *SM*.

**Figure 12:**
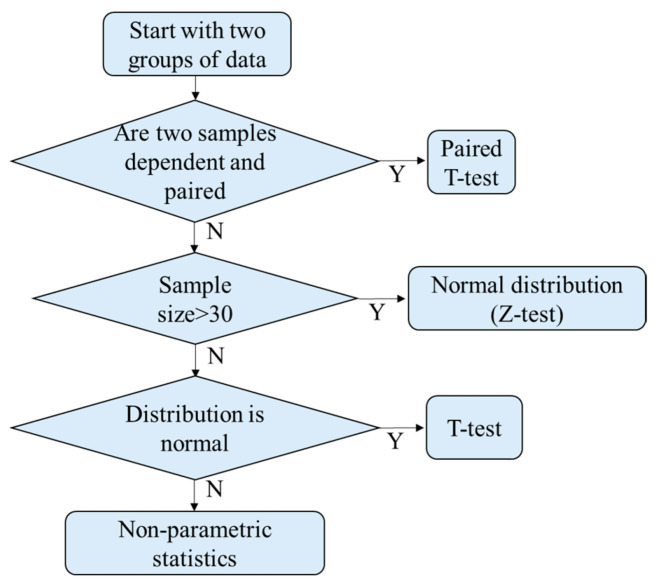
Determine the distribution model.

For further optimization and validation, the data were grouped as training and validation sets. The sample points of the training set and the validation set of *PG* were 6 and 180 points, respectively. The sample points of the training set and the validation set of *AA* were 6 and 210 points, respectively. The sample points of the training set and the validation set of *SM* were 15 and 450 points, respectively. The training set was used to find the optimal threshold using the receiver-operating characteristic curve (ROC) method. The validation set was used to verify the optimized threshold. At first, it is necessary to determine the distribution model of the training set. The process of classification is shown in Figure 12. Statistically, the three bacteria were independent and not paired. Considering that the sample numbers of each type of bacterium were less than 30, the distribution models of the samples were checked for normal distribution using Minitab. If the *p*-value was >0.05, the sample distribution was similar to the normal distribution.

**Figure 13:**
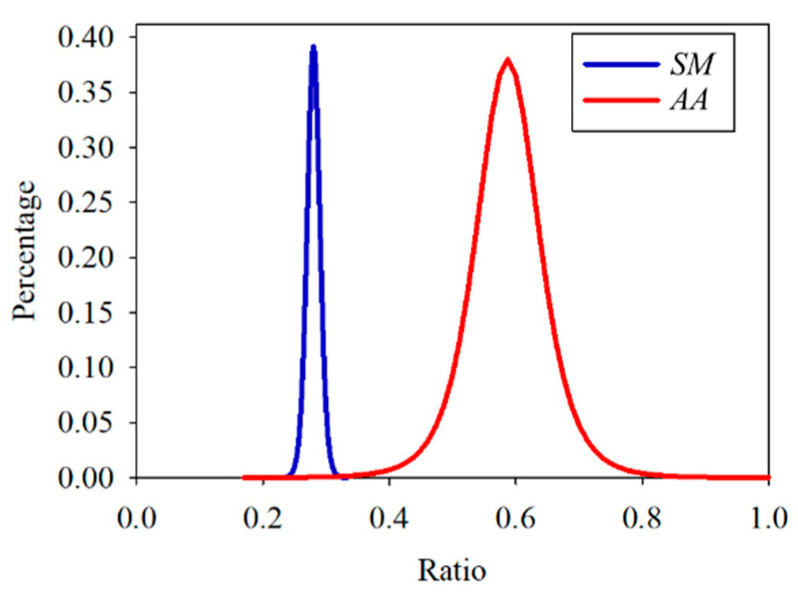
Student’s *t*-distribution of the training set of *Streptococcus mutans* (*SM*) and *Aggregatibacter actinomycetemcomitans* (*AA*).

**Figure 14:**
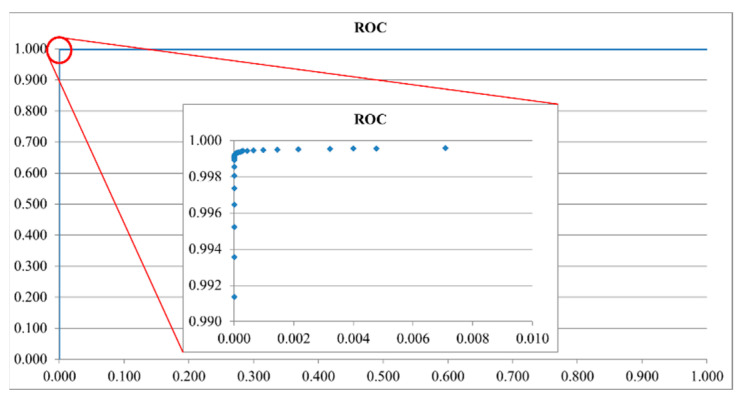
Receiver-operating characteristic (ROC) curve of the training set of *Streptococcus mutans* and *Aggregatibacter actinomycetemcomitans*.

After confirming that the distribution of the three data sets was similar to the Student’s *t*-distribution, each data set was fitted by Equation (5), where Γ is a gamma function, and ν is the sample number minus one. As shown in Figure 13, the ratio value of the cross point of the *SM* and *AA* data distribution curves was about 0.33. According to the ROC curve in Figure 14, the ratio was the optimal threshold for classifying the bacteria as *SM* or *AA*. The sensitivity and specificity of the ratio were over 99%, respectively.

(5) $$f\left(t\right)=\frac{\Gamma \left(\frac{v+1}{2}\right)}{\sqrt{v\pi }\Gamma \left(\frac{v}{2}\right)}{\left(1+t^{2}/v\right)}^{-\left(v+1\right)/2},$$

**Figure 15:**
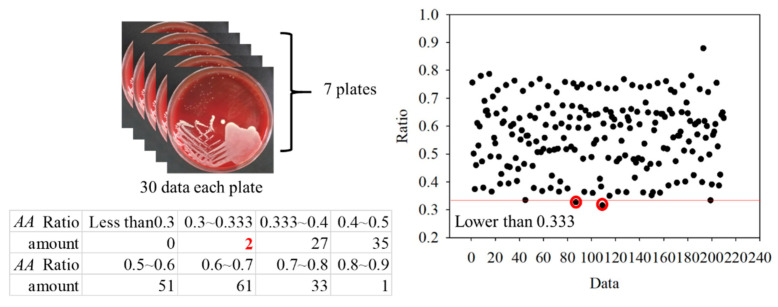
Ratios in the *Aggregatibacter actinomycetemcomitans* (*AA*) groups.

**Figure 16:**
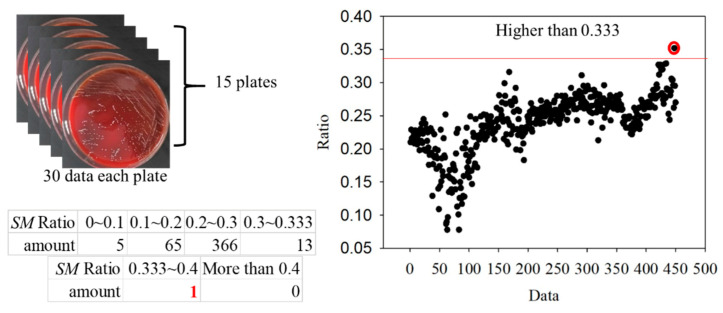
Ratios in the *Streptococcus mutans* (*SM*) groups.

**Figure 17:**
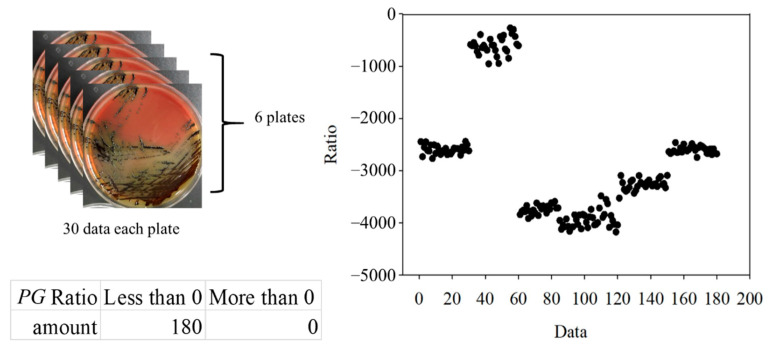
The 500 ± 1-nm data of *Porphyromonas gingivalis* (*PG*) groups.

To further verify the ratio as an optimal identification method, 30 points from each group were used. The data are shown in Figure 15 and Figure 16. In the *AA* groups, only 2 of 210 points were misidentified. In the *SM* groups, only 1 of 450 points was misidentified. The results appropriately fit the evaluation method. As all *AA* and *SM* data at 500 ± 1 nm were positive, the intensities of *PG* at 500 ± 1 nm are shown in Figure 17. The sensitivity and specificity were each 100% because the intensities were all negative. If the intensity of the bacteria at 500 nm was negative, it was classified as *PG*. Otherwise, the ratio of the intensity at 635 nm to a peak value was calculated for the next classification. If the ratio was higher than 0.33, the bacterium was classified as *AA*. Vice versa, if the ratio was lower than 0.33, it was classified as *SM*. This method has a sensitivity and specificity of approximately 99%, respectively.

## 4. Discussion and Conclusions

The fluorescent spectrum of the *PG* under 405 nm excitations had a peak at about 635 nm and a secondary peak at about 705 nm. The result was consistent with the previous studies that found the red fluorescent in *PG* [7,28], since the protoporphyrin IX and coproporphyrin were found in *PG* [21] and the fluorescent spectrum of these porphyrins is similar to that of the *PG* [22,23]. Thus, the fluorescent spectrum of the *PG* could be attributed to the protoporphyrin IX and coproporphyrin. The fluorescent spectrum of the *SM* under 405 nm excitations had a peak at about 500 nm with a long tail extending toward the red wavelength region. This green spectrum was found in relative Streptococci species [7,15,29]. The fluorescent spectrum of the *AA* under 405 nm excitations had a peak of nearly 500 nm and a second peak at nearly 635 nm. The color of this spectrum could be yellow to orange [30]. The fluorescent spectrum of the *SM* and *AA* might originate from flavin adenine dinucleotide and flavins that are the product of bacteria cell energy metabolism [31,32].

The spectral band at 500 nm to 510 nm and 635 nm was used to identify three bacteria species. These spectral bands were also found to be used to detect dental caries and dental plaque in different grades [9,12]. Obviously, the significant spectral bands of fluorescence in cultured bacteria and in-vivo dental plaque were cognate because the autofluorescence might originate from the same endogenous fluorophores, including NADH, flavins, and porphyrins. This study indicated that the fluorescent profiles of these three pathogenic bacteria, *PG*, *AA*, and *SM*, were unique under certain growth conditions and could present a potential risk assessment tool for identifying these key pathogens for periodontal disease or caries. Indeed, the interaction between different oral bacteria is likely to change the fluorescent profile. However, the fluorescent spectrum in a dual-species biofilm could still reflect the presence of *PG* [33]. While uncertainty remains in the case of multi-species biofilms, the ability of fluorescent spectra in bacterial differentiation needs to study further using multi-species biofilms or freshly collected dental plaque combined with microbiome analyses.

In summary, this study comprehensively explored the application of light-induced autofluorescence microscopy to microbiology. First, the excitation light source for the HMS was equipped with narrowband filters. The filters require an OD of at least eight. This helped to eliminate the unwanted excitation light and meet the experimental requirements. Second, the material of the slide was one of the important issues because of the difference in the intensity of the red fluorescence associated with different glass materials. The quartz microscope slides were found to reduce the fluorescence intensity by about 2 dB compared with the borosilicate slide. Third, photobleaching occurred, particularly in *PG*. This phenomenon manifested as an irreversible exponential decay in the fluorescence spectra. Finally, a method for identifying dental plaque bacteria, including *PG*, *AA*, and *SM*, was proposed in this study. The sensitivity and specificity of the method were over 99.8% based on the simulated data, and approximately 99% based on the experimental data. However, fluorescence decay is worthy of further study and improvement. If the bacteria amounts could be quantified and the sample was more uniform, the results would be able to evaluate the consistent *t*_1/2_ of the fluorescence decay.

## Figures and Tables

**Table 1:** Comparison of the plaque disclosing agent and autofluorescence imaging technology in principle, speed, and issues.

	Plaque Disclosing Agent	Autofluorescent Imaging Technology
Principle	Adsorption	Fluorescence
Test duration	5−10 min	Real-time < 1 ms
After the test	Aesthetic issues	No change
Gram-negative bacterial identification	No	Yes
Relative thickness of the plaque	Yes	Yes

## Data Availability

The data presented in this study are available on request from the corresponding author. The data are not publicly available due to their containing information that could compromise the privacy of research participants.
